# Healthcare Provider Perspectives on Digital and Interprofessional Medication Management in Chronically Ill Older Adults of Turkish Descent in Germany: A Qualitative Structuring Content Analysis

**DOI:** 10.3389/fpubh.2022.838427

**Published:** 2022-06-02

**Authors:** Rona Bird, Ilknur Özer-Erdogdu, Meryem Aslan, Hürrem Tezcan-Güntekin

**Affiliations:** ^1^Alice Salomon University of Applied Science Berlin, Berlin, Germany; ^2^Magdeburg/Stendal University of Applied Science, Magdeburg, Germany; ^3^Witten/Herdecke University, Witten, Germany; ^4^University of Siegen, Siegen, Germany; ^5^Berlin School of Public Health, Charité Berlin, Berlin, Germany

**Keywords:** medication management, polypharmacy, interprofessional collaboration, digital health, migration, older adult

## Abstract

Medication management for chronically ill older adults with a history of migration can be associated with specific challenges, for instance language barriers. This study examined healthcare provider perspectives on interprofessional cooperation and digital medication management tools as approaches for increasing medication safety for chronically ill older adults of Turkish descent in Germany. Semi-structured interviews were conducted with 11 healthcare providers, including general practitioners, pharmacists, a geriatric consultant, a hospital social worker, and an expert on digitalization in nursing care. The interviews were analyzed by means of qualitative structuring content analysis. This article presents selected results of the analysis relating to medication management, barriers to optimal medication management, interprofessional cooperation, and digital tools. Compliance was perceived to be high among chronically ill older adults of Turkish descent and the involvement of family members in medication management was rated positively by respondents. Barriers to medication management were identified in relation to health literacy and language barriers, systemic problems such as short appointments and generic substitution, and racism on behalf of healthcare providers. Additionally, the respondents highlighted structural barriers to interprofessional communication in the German healthcare system. Furthermore, two technology acceptance models presented in this article to illustrate the respondents' perspectives on a) a digital application for medication management to be used by chronically ill older adults of Turkish descent and b) a digital tool for interprofessional communication. The discussion highlights the implications of the results for medication management within the German healthcare system.

## Introduction

Mirroring developments within the general population, migrant populations in Germany are subject to the process of demographic aging, with the proportion of people with a history of migration aged 60 and older increasing (1, 2). As of 2018, 2 million of the 19.6 million people with a history of migration living in Germany were aged 65 and older (3)—by 2032 3.6 million people with a history of migration are projected to be aged 65 and older (4).

With advancing age, the likelihood of being affected by chronic illnesses and needing care increases.

Chronic illnesses in old age frequently lead to the concurrent use of 5 or more pharmaceuticals (polypharmacy), which increases the risk of experiencing adverse side-effects (5–8). A further challenge to medication management in Germany is posed by rebate contracts—health insurance providers can enter into contracts with pharmaceutical companies for the purpose of obtaining discounts on particular types of medication. The insured population is then required to accept only the medication produced by the contracted pharmaceutical company if doctors do not certify that the patient must receive the original drug instead of the generic drug. These frequent changes in medication lead to concerns about therapeutic benefits and irritation among patients, thus potentially jeopardizing medication safety (9). While all elderly patients with chronic illnesses and comorbidities are at risk of medication related adverse events, people with a history of migration may be particularly vulnerable, for instance due to language barriers and lower healthcare utilization rates (10, 11). This article presents German healthcare provider perspectives on medication management in chronically ill patients of Turkish descent, emphasizing interprofessional cooperation and digital approaches.

A variety of interprofessional interventions targeting inappropriate prescribing and polypharmacy have been identified in the literature. For instance, specialist geriatric approaches have been implemented to improve pharmaceutical treatment for elderly patients admitted to hospitals (12, 13). Interdisciplinary teams consisting of geriatric specialists, pharmacists, nurses, and other professionals work together to provide assessments, medication reviews, care plans, and consultations to patients aged 65 and older (12, 14, 15). Specialist treatment on an orthogeriatric ward for elderly patients with traumatic injuries has been shown to lead to significantly fewer potentially inappropriate medications being prescribed at discharge compared to usual treatment (15). Additionally, geriatric consultations for trauma patients aged 65 and older have been shown reduce the likelihood of discharge on high- risk medication by 74% in comparison with usual care (13). For elderly patients with comorbid somatic and mental health conditions an interdisciplinary geriatric and psychiatric assessment has been shown to reduce the number of medications prescribed as well as the incidence of potentially appropriate medications at hospital discharge (14). Furthermore, there is some evidence for a beneficial effect of geriatric assessment interventions on the likelihood of patients living at home at 3 and 12 months of follow-up after hospitalization (12) and for positive effects in terms of a reduction of ICU days and earlier discharge, thus making interdisciplinary geriatric assessments cost-effective in relation to usual care (16).

A variety of interprofessional approaches to medication management also emphasize the role of pharmacists in both outpatient and inpatient care settings. For instance, the introduction of pharmacy-based medication reviews, regular dose dispensing and counseling into the primary care regimens of patients with chronic heart failure was shown to improve medication adherence and quality of life (17). The integration of long-term care services, pharmacy services and medical primary care has also been shown to have positive effects on pharmaceutical therapy, with a collaborative community care approach trialed in Slovenia leading to a 50% reduction in clinically relevant drug interactions among geriatric patients taking 10 or more medications concurrently (18).

In a qualitative study conducted in Germany, general practitioners highlighted that they were open to guidance from experienced pharmacists in administering pharmaceutical treatment to elderly multimorbid patients but that integrated care approaches were prevented by systemic barriers such as insufficient channels of communication between healthcare providers (19). In this context, digital approaches could provide a basis for interprofessional communication, for instance by means of video conferences between primary and secondary care providers to coordinate transitions from hospital to home-based care for geriatric patients (20). Furthermore, electronic documentation and patient records in combination with interprofessional medication review processes have been shown to reduce drug-related problems (including drug-drug interactions and adverse drug reactions) (21) as well as potentially inappropriate medication prescriptions (22) in multimorbid geriatric populations. These findings imply that digital approaches facilitating communication with patients and between professionals have the potential to improve prescription practices in relation to elderly patients with chronic illnesses.

**Figure 1:**
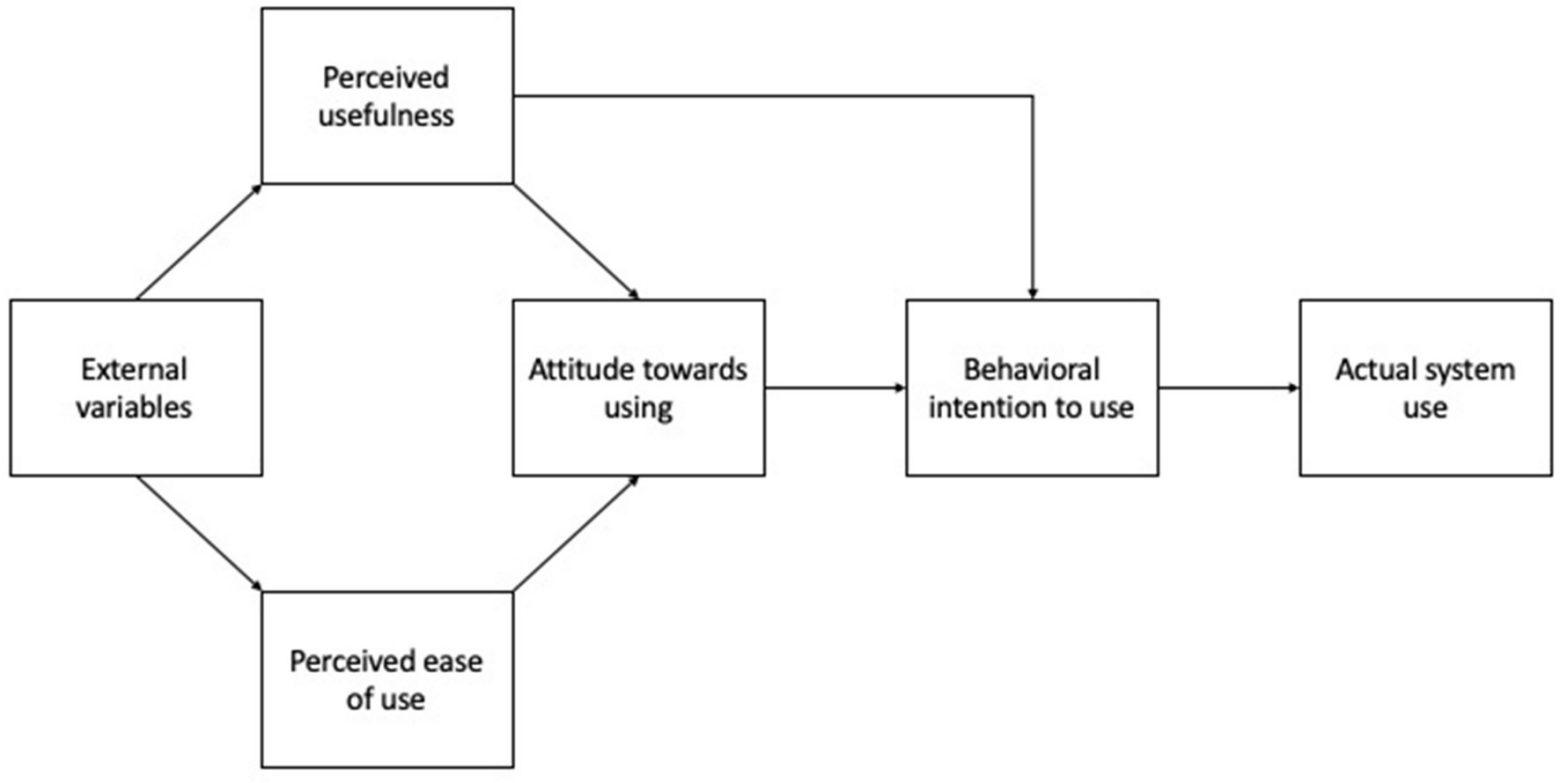
Technology acceptance model (24, 25).

In order to design digital applications that are effective in everyday practice, it is necessary to understand the mechanisms involved in technology acceptance on the patient and provider levels. The technology acceptance model developed by Davis (23, 24) provides a schema that can be empirically evaluated to illustrate the mechanisms involved in the implementation of new technologies in organizational settings. Figure 1 shows the technology acceptance model (25). The most important components, together affecting the additive relationship between “attitude toward using” and “behavioral intention to use” that brings about actual system use, are perceived usefulness and perceived ease of use. Perceived usefulness is defined as “a prospective user's subjective probability that using a specific application system will increase his or her job performance within an organizational context” (25), while perceived ease of use is defined as “the degree to which the prospective user expects the target system to be free of effort.” (25). The model was later elaborated to take into consideration a variety of empirically defined external variables (26). However, for the purpose of this study, the original version of the model was selected as a lens through which to analyze our data because it allows us to define the “external variables” affecting perceived usefulness and ease of use inductively based on our findings.

## Materials and Methods

### Study Design

**Figure 2:**
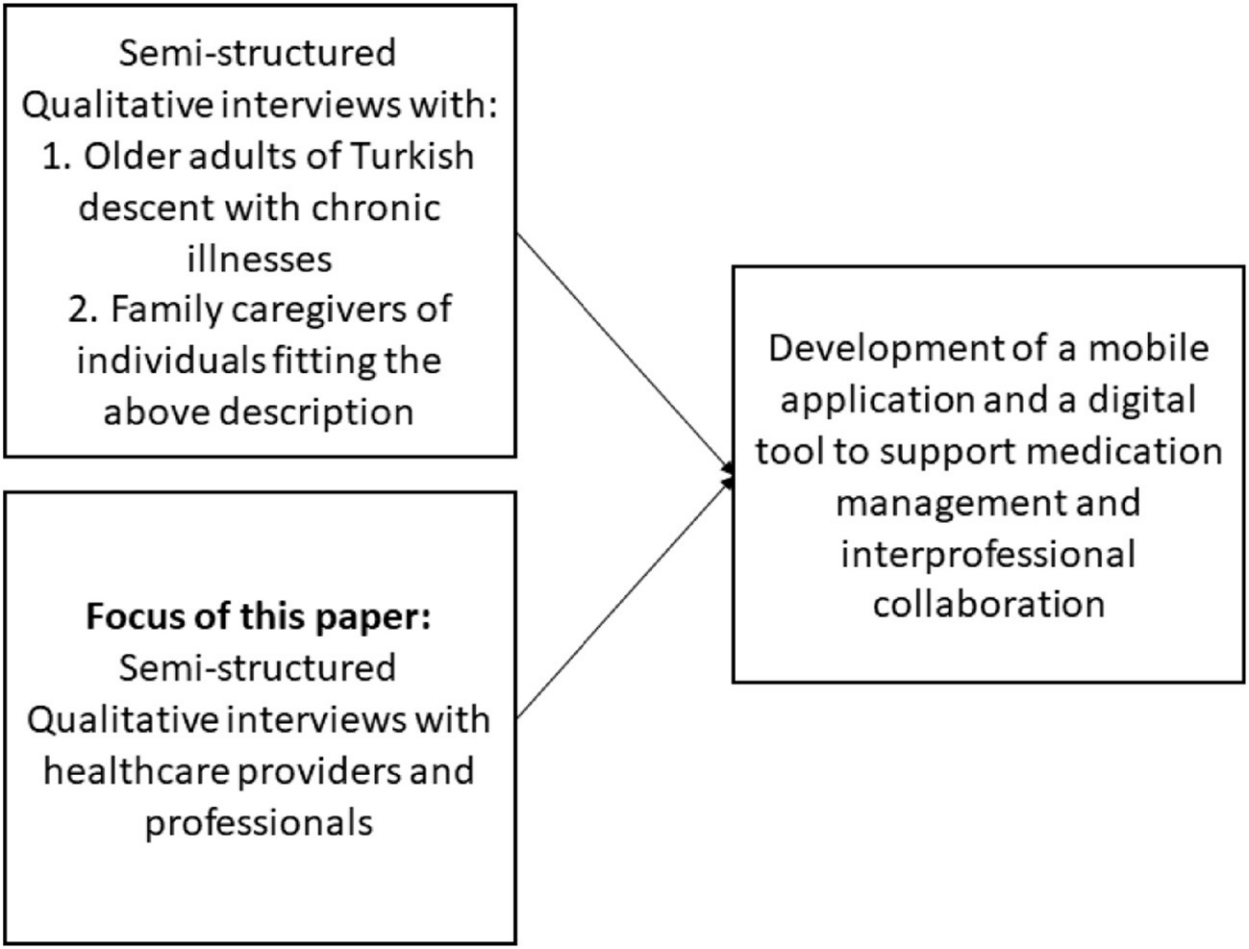
Overview of the three components of the project MedikaMig.

The study presented in this article was conducted as part of a project with three components (see Figure 2):

1) a structuring qualitative content analysis investigating medication management and intake practices as well as attitudes to mobile applications from the perspectives of patients and family caregivers
2) a structuring qualitative content analysis investigating a) prescription and consultation practices and b) attitudes to digital medication management and communication approaches in relation to patients with a history of migration from the perspective of health professionals (presented in this article)
3) the development of a mobile application to improve medication management and a digital tool to support interprofessional collaboration.

A qualitative design was selected to investigate two research questions based on the subjective perceptions of healthcare professionals:

1. How do healthcare professionals navigate and reflect on their prescription and consultation practice in relation to chronically ill patients with a history of migration?
2. Which factors affect practitioners' attitudes regarding digital tools for medication management and interprofessional communication?

The aim of the first research question was to explore how healthcare providers currently perceive medication use among chronically ill patients with a history of migration, how they provide services to this target group and to identify barriers to optimal medication management as well as interprofessional communication. Through the lens of the technology acceptance model (25), these factors can be regarded as the “external variables” (see Figure 1) that influence perceived usefulness and perceived ease of use.

The aim of the second research question was to examine perceived usefulness and perceived ease of use among healthcare practitioners in relation to digital tools for medication management and interprofessional communication. To answer the research questions above, qualitative interviews were carried out and analyzed by means of structuring qualitative content analysis (27).

### Setting and Participants

A total of 11 interviews were conducted −10 interviews were carried out with healthcare providers and one interview with an e-health expert (see Table 1). The participants worked either in Berlin or in Bielefeld in Germany. The inclusion criteria for the health care professionals were: a) working in the healthcare sector and b) frequent contact with chronically ill patients of Turkish descent.

**Table 1:** Overview of participant information.

**Interview partner**	**Profession**	**Nationality**	**Languages**
IP1	Pharmacist	Iranian/German	Persian, German, English
IP2	Pharmacist	Palestinian	German, English, Arabic
IP3	E-health expert	German	German, English
IP4	Geriatric specialist	German, with Turkish migration background	German, English, Turkish
IP5	Psychiatrist	German	German, English, French
IP6	Internal medicine specialist	German	German, English
IP7	Pharmacist	German	German, English, French, Spanish
IP8	Pharmacist	German	German, English, Greek, sign language
IP9	Hospital social worker	German	German, English
IP10	General practitioner	German/Turkish	German, English, Turkish
IP11	General practitioner	German	German, English

### Ethical Data Collection and Handling

Before data collection commenced, an ethics vote was requested from the ethics committee of the Alice Salomon Hochschule Berlin (Ethics vote 05-2019/24). Throughout the data collection process, all participants were informed in writing about the study and the voluntary nature of participation. Informed consent to participate in the study was obtained in writing. All data were handled in accordance with German data protection regulations.

### Data Collection

Semi-structured interviews were conducted using an interview guide. The interview guide covered the following topics:

- Health status of chronically ill patients with a history of migration from Turkey
- Medication use and compliance
- Barriers and resources for medication management
- Desires and expectations in relation to pharmaceutical treatment expressed by patients and relatives
- Interprofessional collaboration
- Digital tools
- Suggestions for better medication management.

### Data Analysis

The interview data were analyzed using structuring qualitative content analysis according to Mayring (27). The analysis started with the development of a system of categories and subcategories to be deductively applied to the data. The categories were developed based on the interview guide and were refined by means of an initial inductive analysis of two of the interview transcripts. During the further analysis steps, inductive categories could be added.

**Figure 3:**
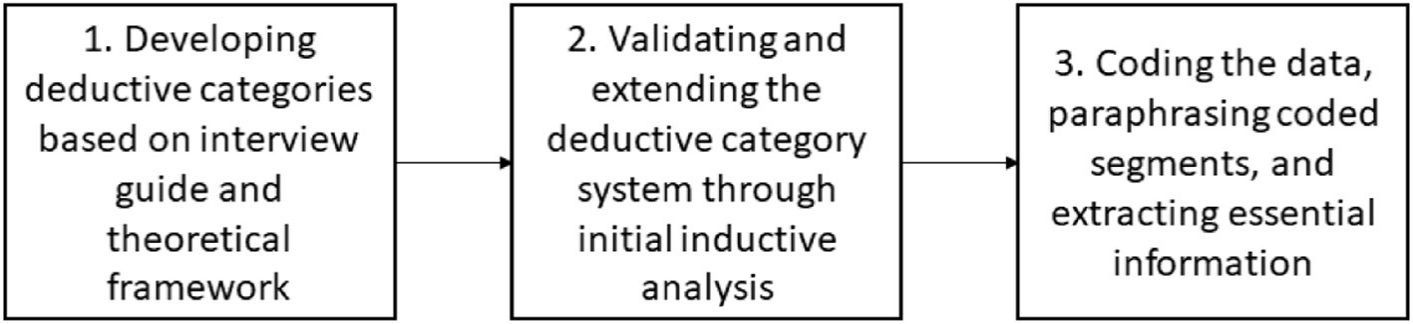
Summary of the steps involved in qualitative structuring content analysis.

Each interview was coded according to the system of categories. Each coded section of interview text was then paraphrased and subsequently the most essential information from each section was extracted through a second step of abstraction. In order to ensure reliability, the analysis steps were carried out independently by two researchers (RB and MA). Disagreements were discussed in the large team until a consensus could be reached. Figure 3 summarizes the steps involved in the data analysis.

A total of 7 categories and 24 sub-categories were developed and applied to the interview data (Supplementary Material 1)

This article will focus in particular on the following categories:

- Medication management
- Barriers to optimal medication management
- Interprofessional cooperation
- Digital approaches to medication management and interprofessional communication.

The categories above and selected sub-categories will be presented below to answer the research questions.

## Results

### Medication Management

Prescription and consultation practices from the perspective of healthcare providers will be outlined below in the context of the practitioners' perceptions of medication management within the target group. Additionally, barriers to optimal medication management and perceptions of interprofessional cooperation are outlined below as the backdrop against which professional prescribe and consult chronically ill patients with a history of migration. The categories presented below can be understood to represent “external variables” influencing perceived usefulness and perceived ease of use in a technology acceptance model.

#### Healthcare Provider Perspectives on the Utilization of and Attitudes to Medication Among Elderly Migrants With Chronic Illnesses

While professionals noted that prescriptions were obtained regularly by chronically ill patients of Turkish descent, Perceived patterns in medication use also included risky tendencies such as taking medication based on recommendations from friends and family members (Interview Person (IP) 2, line 268–269, IP10 361–366), a reluctance to take medication if not for family members monitoring intake (IP4 140–147), and the misconception that medication intake can always be increased or reduced in response to changes in symptoms (IP10 24–29). Assistance during medication intake was deemed most likely to come from family members (IP4 152–156). The regularity of medication intake was difficult for some professionals to judge (IP7 24–25, IP9 48–52) but professionals largely agreed that chronically ill patients of Turkish descent seemed to take their medication on a regular basis as prescribed (IP 5 25 & 33, IP6 28–32, IP8 36–42). However, old age was perceived to interfere with the regularity of intake regardless of a history of migration (IP7 57–59).

Perceived attitudes to medication among chronically ill patients of Turkish descent varied according to different professionals. IP4, a geriatric specialist, reported that some patients do not view their medication as helpful and therefore do not consider it necessary to take them (IP4 140–147). On the other hand, IP10, a general practitioner, stated that medication generally has positive connotations among elderly patients of Turkish descent (IP10 125–133).

##### Prescription and Consultation Practices in Relation to Elderly Migrants With Chronic Illnesses

Patient education was perceived to be understood more effectively when carried out in the presence of family members (IP8 70–72). Pharmacies were considered an essential port of call for patient education regarding dosage, timing of medication intake, the effects of prescribed medication, and side effects (IP10 37–40). According to IP10, physicians face challenges in providing effective patient education to chronically ill patients of Turkish descent, for instance in the form of time pressure (IP10 37–40), which makes consultations particularly difficult when patients require repeated explanations to understand the mechanisms underlying their chronic conditions (IP10 114118, IP10 163–165) or when patients lack language competencies (IP7 147–150, IP10 170–179). Socioeconomic status was also highlighted as a barrier to optimal patient education and medication management “language is the main barrier, but social status is also important because if someone has a limited understanding about how the body works and how illness works, they can't follow.” (IP10 170–172). These constraints were perceived to be particularly challenging for healthcare providers when explaining the importance of medication for chronic conditions, for instance hypertension, which does not always cause symptoms, but requires continuous pharmaceutical therapy:

“and if someone has hypertension, the kinds of consequences that can lead to, one has to keep educating the patient over and over again and explain that it doesn't hurt and they might not notice it, but it can keep causing damage” (IP10 114–116).

#### Barriers to Optimal Medication Management

##### Barriers on the Level of the Healthcare System

The respondents highlighted a lack of provisions for linguistic and cultural diversity in the healthcare system (IP3 70–75), which was perceived to increase mistrust toward institutions and professionals on behalf of elderly migrants (IP4 246–252, IP 10 154–155). Subsequently, utilization of services such as home-based care services was perceived to be significantly lower among elderly migrants in comparison to the general population (IP3 75–80).

Several respondents highlighted that older adults with a history of migration, in particular those subject to language barriers, were particularly vulnerable to the effects of resource scarcity in the healthcare system, for instance in terms of timely access to primary care and adequate medication conciliation (IP2 99–103, IP2 147–151). IP1, a pharmacist, highlights how, given the limited amount of time available for medication conciliation during general practice appointments, doctors should delegate more to pharmacists:

“the doctors have, you hear it from every patient, they barely have time. They are stressed themselves and the patients suffer as a result and they have to acknowledge that they can't do everything themselves. The doctors can't diagnose, care for the patient well and at the same time do a medication analysis. […] It's not that they don't have the potential or the professional competencies, they just don't have the time” (IP1 181–187).

Staff shortages and short time slots for appointments were also emphasized as being particularly damaging to elderly migrants with chronic illnesses, who might require longer consultations to ensure trust-building and linguistic comprehension (IP2 17–19, IP7 159–165). The pharmacists in the sample also identified economic barriers to optimal medication management, for instance highlighting that certain pharmacy services such as medication conciliation appointments have to be paid for by patients themselves (IP1 199–204). Additionally, several respondents referred to problems caused by generic substitution practices in the context of rebate contracts of health insurance providers with pharmaceutical companies. As a result of these types of contracts, chronically ill individuals frequently see changes in their medication in the form of generic substitution. For elderly migrants, who may struggle to keep track of instructions for use and purpose of their medication due to language barriers, this can cause significant distress, which is in turn passed on to healthcare providers (IP8 95–97; IP2 157–161). IP 10 describes the problem:

“because of these contracts, a terrible invention, the color changes. They [older adults with a history of migration] organize their medication by color, so when they come to me they don't say I took Losartan, they say I took the pink colored one, the pink one. So that's how the medication is described and then if it's no longer pink, it's white, they mix it up with another betablocker” (IP10 45–50).

##### Barriers on the Individual Staff Level

Barriers to optimal medication management extend from the structural to the individual level, with several participants reporting incidents of racism toward older adults of Turkish descent in the healthcare system (IP4 335–348, IP7 122–124). In fact, one of the respondents in the study reported holding prejudiced views themselves, stating:

“It's exhausting. And in my everyday life at work- I'll give you an example. Let's say a female patient of Turkish descent comes in and she's quite hysterical – in my perception. Then it would take me ten minutes to first of all talk her down from that state of hysteria. That's really a lot of work for me. And when I feel stressed, it makes me aggressive” (IP11 294–298).

While qualifications and training were seen by other participants as valuable resources in increasing awareness and competencies among practitioners regarding diversity sensitivity (IP1 216–222), IP 11 also state that they have no interest in utilizing training opportunities, citing an “aggressive psychological barrier” as a justification (IP11 288–292).

#### Interprofessional Cooperation

##### Barriers to Cooperation for Medication Management Between Professionals

The participants mentioned multiple barriers preventing effective interprofessional communication and collaboration in relation to medication management for older adults with a history of migration. A lack of professional recognition was perceived from a pharmacist perspective, stating that doctors do not realize how important the role of community pharmacies can be in ensuring medication safety (IP1 180–181). Perceptions about other professional groups were also shared by doctors, on the one hand by a general practitioner complaining about a lack of respect from pharmacists (IP6 107–114) and on the other hand by a geriatric consultant complaining about general practitioners not following instructions from specialists (IP4 397–399). When considered in the context of assertions defining the German health care system as fragmented and lacking any existing structures for interprofessional communication (IP 8 188–190), these complaints about other professional groups may be understood to reflect frustration resulting from practitioners having to interpret the decisions of other professionals, possibly made under time pressure, without the option of communicating directly. In a minimally digitalized healthcare system, doctors and pharmacists primarily communicate by means of telephone, with doctors' practices generally not having separate lines for patients and professionals (IP2 126–130). Several participants highlight that the German healthcare system is not designed for interprofessional cooperation, with strictly separated care settings and fragmented care transitions (IP3 114–118, IP4 459–463, IP9 426–428). IP8 highlights:

“I think it's partly still a system that was not designed for cooperation, so maybe even the whole healthcare system including the health insurance providers, actually everyone is just trying to do their own thing, even though, if we worked together, we could maybe accomplish a lot more” (IP8 188–190).

##### Conditions for Successful Interprofessional Cooperation

Frequent and respectful communication within teams of the same organization, for instance in the hospital sector (IP9 301–305) and between professionals based in different care settings, for instance general practice and pharmacies (IP1 188–190), are perceived as requirements for successful interprofessional collaboration. Additionally, clear written communication is highlighted by participants as a particularly important aspect of interprofessional collaboration in the German context, where professionals in different care settings rarely have the opportunity to communicate directly. Some participants emphasize that comprehensive documentation of prescription decisions can allow other professionals to understand the rationale behind changes to the medication regime and can thus help ensure medication safety for patients (IP4 432–436, IP7 203–207).

### Digital Approaches to Medication Management and Interprofessional Cooperation

Within the following section, two technology acceptance models will be developed. The “external variables” outlined above in answer to the first research question will be shown in relation to the perceived ease of use and perceived usefulness identified for a mobile application for medication management and a digital tool for interprofessional communication in two technology acceptance models.

#### Medication Management Application for Chronically Ill Patients of Turkish Descent

**Figure 4:**
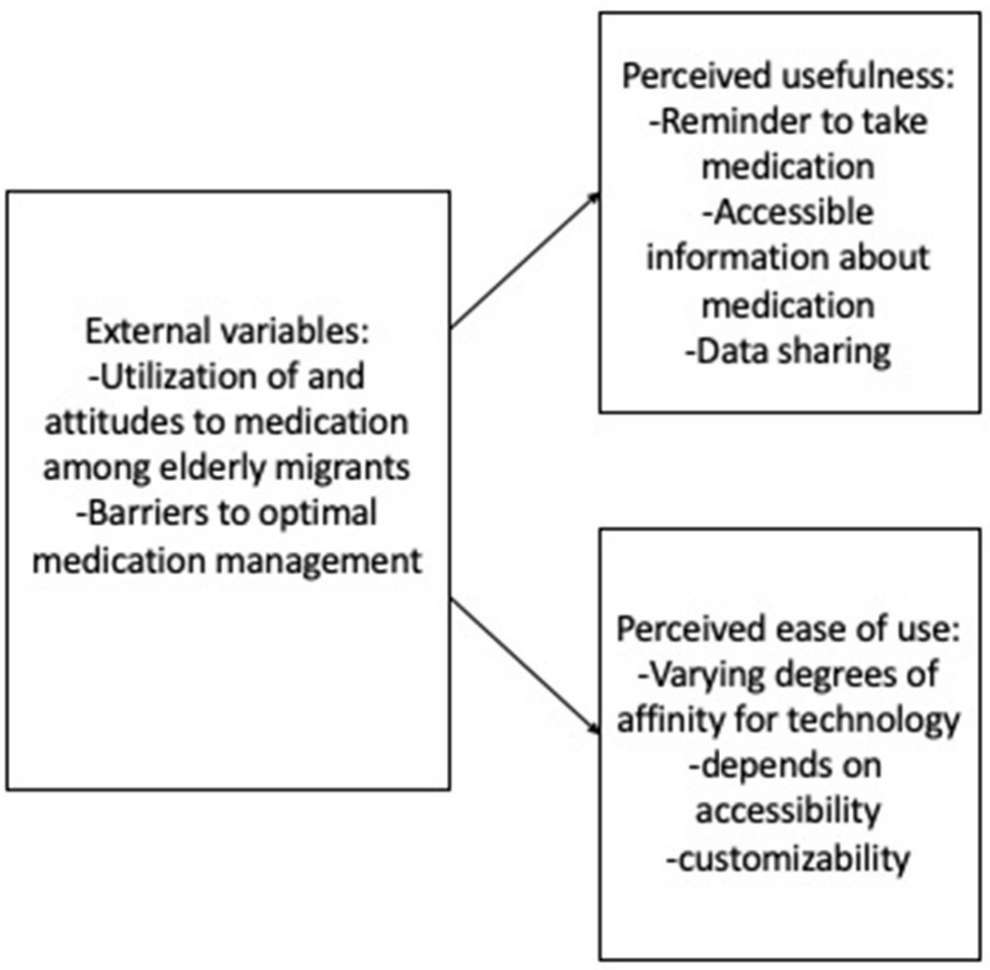
Factors playing a role in technology acceptance (24, 25) for a mobile medication management application for older adults of Turkish descent with chronic illnesses.

Figure 4 below represents how the external variables relating to medication management and barriers to optimal medication management feed into perceived ease of use and perceived usefulness from the perspective of healthcare providers. The practitioners' understanding of the target group and the barriers they face in relation to medication safety affect their recommendations for increasing ease of use and usefulness as outlined below.

While participants agreed that the use of a mobile application for medication management requires a certain level of affinity for digital technology, they disagreed on the prevalence of this affinity among older adults of Turkish descent. Two of the participants drew on their own experiences, with IP4, a geriatric consultant of Turkish descent, highlighting that several of his family members in their late 50s were highly proficient at using mobile applications IP4 (612–622). Conversely, IP1 drew on her experience to highlight:

“I'm from Iran myself, that's one of Turkey's neighboring countries, and when I think of my grandmother, or her generation, they don't like that sort of thing. They prefer to receive information via phone calls […] to inform themselves in a digital way, I don't see any openness” (IP1 100–104).

In terms of the perceived usefulness of a potential medication management application for older adults of Turkish descent with chronic illnesses, many of the participants agreed on three core attributes. First, several participants emphasized the advantages of setting up notifications reminding participants to take medication regularly (IP1 117–118, IP2 179–184, IP4 550–557, IP5 133–135, IP6 145–146, IP9 67–70). Second, the respondents stated that the app should provide an overview of information relating to prescribed medication in the user's native language, including instructions for intake (IP5 128–130) and information about potential interaction effects (IP1 123–124). Third, a medication management application was seen as an opportunity for data sharing. On the one hand, this could facilitate communication between patients and healthcare providers, for instance by allowing the sharing of data about regularity of intake (IP2 241–245, IP3 206–215, IP4 567–576) or about medication availability so that new prescriptions can be prepared quickly and efficiently (IP9 394–400). On the other hand, relatives could also benefit from easily accessible intake data, for instance if they are responsible for medication management as family caregivers (IP4 566–567). Furthermore, regarding perceived ease of use, the professionals emphasized several design aspects that should be taken into consideration to make a medication management application accessible to older adults of Turkish descent. Aside from making the application available in the users' native languages as well as in simple language (IP3 246–251, IP5 128–130, IP6 163–168), the participants emphasized accessibility for individuals with visual or hearing impairments (IP7 220–223, IP4 581–585). Additionally, a high degree of customizability was emphasized, considering that the Turkish community in Germany is highly heterogeneous (IP9 375–381) and attempts to cater to a specific stereotype through design choices should be avoided, as explained by IP 4:

“I think it's important to find out, to understand what the patient prefers and not make it too ridiculous. For example, I don't know, using Allahu Akbar as a reminder to take medication, I think that's ridiculous” (IP4 550–557).

#### Digital Tools to Facilitate Interprofessional Communication

The participants on the whole expressed that they considered digital tools to communicate with other professionals to be useful. The central function desired by the participants was that of data sharing. Several participants proposed a digital platform where different professionals, for instance doctors and pharmacists, can access patient data relating to diagnoses and prescriptions (IP2 230–235, IP3 92–92, IP9 428–432). Additionally, interest was expressed in digital means of communication between professionals, for instance in the form of an encrypted chat platform, so that information can be exchanged quickly without using the telephone (IP8 199–209, 234–240). The main parameter in relation to ease of use highlighted by the respondents was a user interface that is integrated into existing (software) infrastructure (IP3 292, 360–363, IP9 428–432). As well as expressing the desire to integrate any new tools into existing software, IP9 (225–228) suggests that QR codes that are already found on medication plans in Germany could be used more effectively within digital tools for interprofessional communication. The main barrier perceived in relation to the use of a digital application for interprofessional communication was data protection legislation. This significantly lowers the perceived usefulness of digital tools for interprofessional communication. The participants highlighted that current data protection regulations make it impossible to share patient data digitally (IP2 204–210, IP8 249–253, IP9 463–469). IP 9 highlights that healthcare providers are currently struggling to transition from fax to email as a medium of communication:

“how can we exchange information in a way that complies with data protection regulations without having to type it up all over again, of course I can fax it, but that will eventually be phased out, so that's what everyone is preparing for now, how can we use email in a way that complies with data protection regulations throughout the entire healthcare system, for example I don't know if a GP has an encryption system” (IP9 455–463).

Even plans to make patient records available digitally by saving the data on individual health insurance cards have not yet come to fruition in Germany, so an application for data sharing like the one envisioned by the respondents is unlikely to be able to be implemented (IP7 248–255). Given these restrictions, some participants emphasize other aspects of digital infrastructure, such as encrypted email, as a means of improving channels of communication in the healthcare sector (IP9 455–463). Thus, while the respondents perceived digital tools for interprofessional communication as useful in theory, the usefulness in practice was deemed low due to restrictive data protection regulation and a lack of digital infrastructure.

## Discussion

**Figure 5:**
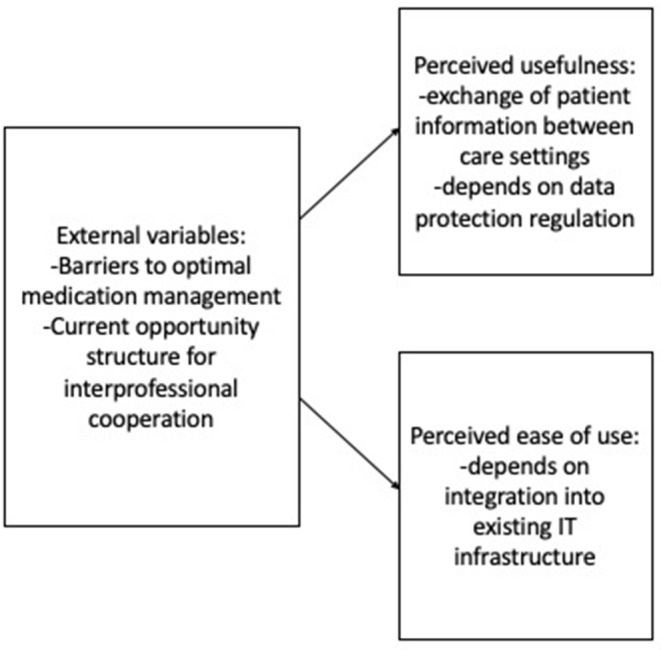
Factors playing a role in technology acceptance (24, 25) for a digital tool for interprofessional communication.

The present study explored how German healthcare providers perceive medication management among chronically ill older adults of Turkish descent, which barriers they perceive to optimal medication management, and how interprofessional cooperation is currently perceived. These factors can be understood to represent “external variables” (TAM) in technology acceptance models for a mobile application for medication management (Figure 4) and for a digital tool for interprofessional communication (Figure 5). Additionally, the study presents perceived ease of use and perceived usefulness for the aforementioned digital technologies. Given the qualitative nature of the study and the small sample size, these findings cannot be generalized to the German healthcare context as a whole. However, they provide explorative insights into possible factors shaping healthcare providers' attitudes and practices in relation to (digitally supported) medication management among chronically ill older adults of Turkish descent.

With regards to provider perceptions of medication management among chronically ill older adults of Turkish descent, the present study suggests that compliance is rated positively in the target group. However, language barriers and low socio-economic status, which are among the conditions of low health literacy, are perceived as barriers to patient education (10, 28, 29). Further barriers to optimal medication management on the level of the healthcare system are shortages of time and staff. Generic substitution was also perceived to threaten medication safety, as pointed out by Gradl et al. (9). With regard to interprofessional cooperation, the present study confirms the findings of Löffler et al. (19), which suggest that German healthcare providers perceive systemic barriers that prevent effective communication between professionals. The previous findings on medication management primarily refer to interprofessional cooperation between professionals in one setting, such as geriatrics (12–16), which is already oriented toward interdisciplinary cooperation. Our findings indicate that increased intersectoral collaboration in the German healthcare system requires bridging to existing structures – variations in resource availability, workflows and communication infrastructure between sectors need to be addressed. Digital tools enabling instant communication could allow for more integration between sectors. Further studies on intersectoral interprofessional care are needed to understand the conditions of successful interprofessional cooperation.

The analysis has highlighted conflicts between professions that make interprofessional communication difficult. The cause of these conflicts would need to be further investigated through qualitative hermeneutic-reconstructive studies. A possible avenue for interventions could be the development of interprofessional training courses on polypharmacy so that different healthcare providers can meet on an equal footing, thus allowing for a better understanding of other professional perspectives.

While digital tools to improve interprofessional communication were regarded positively by the participants of this study, particularly as a way of exchanging patient data between care settings, the perceived usefulness was low overall due to restrictive data protection legislation. Perceived ease of use was found to hinge predominantly on integration into existing IT infrastructure in the healthcare sector.

A mobile application to support medication management among older adults of Turkish descent was overall rated positively by the participants of this study. Suggestions were made to optimize perceived usefulness by means of reminding, information, and data sharing functions. Healthcare providers regarded accessibility factors and customizability as particularly important factors, given the assumption of significant variety in technology affinity among the target group. Intervention studies should investigate whether this type of web application could lead to a reduction in irritation and rejection of generic drugs among older people, especially migrants, who have difficulties in acquiring and applying new health knowledge due to low health literacy. The aim would be to find out what impact these apps have on adherence among this target group.

The central limitation of this paper is the exclusive focus on healthcare provider perspectives. For the technology acceptance model pertaining specifically to digital tools for interprofessional communication, this is less of a limitation, since healthcare providers are the target group for this technology. However, the technology acceptance model relating to the medication management application for chronically ill older adults of Turkish descent should be regarded as incomplete at this stage. In the future, it will be augmented from the perspective of chronically ill older adults of Turkish descent and their family caregivers based on the other two studies conducted within the project MedikaMig. Another limitation is the small selection of experts from the respective disciplines, which provides a valuable insight into the field, but could be expanded, for example, in a maximum contrasting further involvement of experts in the study and the acquisition of experts from rural regions. Finally, while the findings of this study provide insights into subjective perceptions of healthcare providers on medication management in older adults of Turkish descent and the use of digital support, they should not be interpreted as being generalizable to other contexts.

## Data Availability Statement

The raw data supporting the conclusions of this article will be made available by the authors, without undue reservation.

## Ethics Statement

The studies involving human participants were reviewed and approved by the Ethical Committee of the Alice Salomon University of Applied Science Berlin. The patients/participants provided their written informed consent to participate in this study.

## Author Contributions

The authors HT-G and IÖ-E developed the project idea, wrote the project proposal and managed the project and were involved in the conception of the data collection instruments, and the data analysis. RB and MA worked on the conception of the data collection instruments and the data analysis. RB and HT-G conducted the expert interviews. RB was the lead author of the paper and all other authors made relevant contributions in different stages of preparing the paper. The paper was finalized jointly by all authors. All authors contributed to the article and approved the submitted version.

## Funding

The study reported in this article was conducted as part of the project MedikaMig: Vermeidung von Polypharmazie bei Menschen mit Migrationshintergrund and was funded by the Institut für angewandte Forschung Berlin (IFAF) between 2019 and 2021.

## Conflict of Interest

The authors declare that the research was conducted in the absence of any commercial or financial relationships that could be construed as a potential conflict of interest.

## Publisher's Note

All claims expressed in this article are solely those of the authors and do not necessarily represent those of their affiliated organizations, or those of the publisher, the editors and the reviewers. Any product that may be evaluated in this article, or claim that may be made by its manufacturer, is not guaranteed or endorsed by the publisher.

## Supplementary Material

Supplementary Material 1 Categories and subcategories.
