# Supplementary material for: Healthcare Provider Perspectives on Digital and Interprofessional Medication Management in Chronically Ill Older Adults of Turkish Descent in Germany: A Qualitative Structuring Content Analysis
Source: Front Public Health. 2022 Jun 2;10:838427. doi: 10.3389/fpubh.2022.838427 (PMC9201245; doi:10.3389/fpubh.2022.838427)
Supplement: Supplementary Material 1 — Categories and subcategories. [file Table_1.pdf]

Digital and Interprofessional Medication Management – Bird, Özer-Erdogdu, Aslan, Tezcan-Güntekin
Supplementary Material: Categories and Subcategories

- 1. Health conditions
  - 1.1. Health conditions experienced by elderly migrants of Turkish descent
  - 1.2. Comparisons between patients of Turkish descent and patients without migration backgrounds
  - 1.3. Healthcare utilisation among elderly migrants
- 2. Medication
  - 2.1. Medication taken by elderly migrants
  - 2.2. Perceived extent of polypharmacy (taking more than 5 different types of medication concurrently) among elderly migrants
  - 2.3. Utilisation of- and attitudes to medication among elderly migrants
  - 2.4. Prescription and consultation practices
  - 2.5. Gender differences in medication use
  - 2.6. Evidence and consequences of polypharmacy
- 3. Expectations in relation to medication
  - 3.1. Expectations held by patients
  - 3.2. Expectations held by family members
- 4. Barriers and resources
  - 4.1. Barriers on the societal level
  - 4.2. Barriers on the healthcare system and organizational/institutional level
  - 4.3. Barriers on the personnel level
  - 4.4. Barriers on the patient level (including family members)
  - 4.5. Support needs
  - 4.6. Resources/coping strategies
- 5. Interprofessional cooperation
  - 5.1. Successful interprofessional cooperation
  - 5.2. Poorly functioning or absent interprofessional cooperation
- 6. Web-Application
  - 6.1. Requirements for an application serving elderly migrants
  - 6.2. Requirements for an application aiming to improve interprofessional cooperation
  - 6.3. Challenges for an application serving elderly migrants
  - 6.4. Challenges for an application aiming to improve interprofessional cooperation
- 7. Potential for improvement: Existing approaches, examples of good practice
